# The Story of the Hospitals

**Published:** 1887-01-15

**Authors:** 


### The Story of the Hospitals.

(*Continued from page* 212.)

### The British Home for Incurables.

Helpless. Hopeless. Homeless.

The pre-occupied City man as he passes up and down the Clapham Road on his way to and from his business, has probably become so accustomed to every detail of the thoroughfare, that he scarcely spares a thought for the modest row of houses wherein the British Home for Incurables carries on its work, but no stranger visiting the neighbourhood can fail to be struck with the neat and cheerful appearance of the Home, standing back from the road, and bearing on its walls, in addition to its title, the inscription, "Supported by Voluntary Subscriptions." It would be hard, indeed, for people who have never seen Clapham not to know something about the Charity, for its claims to the support of the benevolent public are set forth in a variety of ways, which do credit to the business-like aptitude of the secretary, Mr. R. G. Salmond. "Helpless, Hopeless, Homeless," is an alliterative appeal which must be well known to every reader of the newspapers, and, if they miss the announcement, they may be sure, if their names are to be found in either the London or the Suburban Directories, they will be reminded generally about Christmas-time, when purse-strings are supposed to be the least tightly drawn, that there is such an institution as the British Home for Incurables. And, indeed, it is hard to imagine an appeal which can come with more force than this in aid of a class upon whom the burden of affliction has been laid most heavily. There is a Rabelaisian proverb which declares that "very small rain lays a great deal of dust," and the managing head of this Home has evidently taken it to heart.

The Small Rain and the Dust.

Anxious at all times for the support of the rich and the well-to-do, never losing an opportunity of putting in a word in its own cause when charitable people are thinking of bestowing substantial gifts, yet the British Home is endeavouring to demoralize benevolence—if the term may be used. The same principle, of course, is at the bottom of the Hospital Saturday and Hospital Sunday funds, but the Clapham Road Institution affords the most striking instance of which we know of the success of the system as applied to an individual charity. It would not be a dangerous task to undertake to prove that the lower you descend in the social scale the wider and deeper and purer does the stream of philanthropy flow. That the widow's mite is a more acceptable offering than the munificent donation of the wealthy we know on Divine authority, and the postal order for five shillings which comes from a lower middle-class household is sometimes an offering of truer charity—because it represents greater self-denial—than the hundred pounds cheque of the millionaire. Do not let it be supposed that we desire to check the flow of benevolence, which the wealthy, to their honour be it said, are constantly feeding. There are suffering and misery enough in the world to call for the utmost exertions of the sympathetic of every class. Still the comparative success which has attended these appeals to the general public on behalf of the British Home for Incurables suggests the thought that perhaps, after all, the managers of our charitable institutions have not sought the co-operation of the multitude as they might have done. Not many months ago Mr. Salmond issued a modest demand on all and sundry for a million shillings—he has already received nearly six hundred thousand of them.

200,000 Incurables die annually.

Homes for incurables are quite a modern development of the philanthropic spirit. This was one of the earliest, yet it has only this year completed a quarter of a century's existence. It might have been thought that the sad case of these poor people, of whom it is computed that no less than 200,000 die every year, would have moved the hearts of the charitable long ago. How many incurables still look, like Queen Katherine, upon a world "where no pity, no friends, no hope, no kindred weep for me," the yearly list of candidates on the books of the British Home only too plainly show. This institution, once established, has grown apace, and though there have been times when the pinch of poverty has been sorely felt, yet on the whole its record is a prosperous one. In truth, the work which it does appeals so directly to human sympathy that it is inconceivable that any serious contraction of its beneficence should ever be necessitated.

Like other charities, its funds are not commensurate with its full needs, and one or two empty rooms in the Clapham Road establishment are mute witnesses to the fact. Still, the British Home for Incurables has much to be thankful for. In the first place it has succeeded in attracting, to a peculiar degree, the attention and patronage of the Royal family. Two years after its establishment, the Princess of Wales, who

The Princess of Wales.

had just come to England as the bride of the heir-apparent, had her notice directed to this small and struggling charity, as it was then. With that impulsive sympathy which has always been a characteristic of Her Royal Highness, the Princess at once became the patroness of the Home, and gave a handsome donation. The impulse was not merely an evanescent force, for ever since the Princess has taken the liveliest interest in the welfare of the institution. Other donations have been received from her hand, she has paid a private and a public visit to the institution, and has imbued other members of the Royal family with her zeal in the cause of the Home. Among the sights of the Home which no visitor is allowed to miss, are copies of the two books of the Queen, *Leaves from the Journal of our Life in the Highlands*, and *More Leaves*, inside each of which is written, in the bold and eminently characteristic hand of Her Majesty, "Victoria R. and I."

Persons for whom the Home is not Intended.

In a former paper much was said of the history of the Home for Incurables, and a word as to the actual class of patients for whose relief it is intended will not be out of place. In the first place it should be observed that persons of the class to whom parochial relief is given are not admitted either as in-patients or out-patients, for the very simple reason that these will receive adequate attention in the Union infirmaries. In the next place, idiots or imbeciles, sufferers from cancer, epilepsy, or any other disease which necessitates isolation, do not come within the scope of the work of the Home itself, although out-pensions are granted to people afflicted with the two last-named diseases. Most of the inmates belong to the middle classes, and are sufferers generally from paralysis and rheumatism, or both, spinal and hip diseases, and affections of the heart and lungs, when these are complicated with some other ailment which renders the patient wholly or partially incapable. People of this class of life, and in such a condition, are indeed pitiable objects. They have known comfort, if not luxury, and are too morbidly sensitive to publish their woes. If they have friends, they know that those friends can ill-afford the constant drain which the support of a confirmed invalid makes upon the domestic exchequer; and if they have none, as is often the case, they suffer in silence, existing perhaps on a small annuity, the sole remainder of other and happier days, eked out with the proceeds of needlework, each stitch of which represents an added pang to their sufferings. Most of these forlorn ones could say of themselves, in the words of a Shakespearian heroine, "Alas, I am a woman, friendless, hopeless,"—for the weaker sex largely predominates on the books of the charity. No candidate, we should add, is received into the Home at an earlier age than thirty-five; and admission is obtained by the

No Help from Saturday or Sunday Fund.

election of subscribers. It is because this "voting system," as it is generally called, prevails here that the Home is debarred from participation in the collections made on Hospital Saturday and Hospital Sunday. The decision of the managers of these funds as to the class of charities which shall be benefited by the money at their disposal has, no doubt, been arrived at after full consideration, and perhaps, on broad grounds, its justice is incontestable. It is difficult, however, to see on what other system the British Home for Incurables could be conducted, for the admissions to the institution and the out-pensions are alike charges for life. There is no coming and going of patients as in an ordinary hospital. The subscribers may very fairly claim a voice in the election of the recipients of their bounty. Provided, therefore, candidates can ensure election, there is no limit to the number of out-pensioners, and practically no limit to the number of in-patients, except that which is marked out by the extent and amount of public subscriptions. "The charity"—here we quote from the documents of the Society itself—"extends its benefits to all parts of the United Kingdom, and all who are deemed incurable, and are of the class for which it is designed, will be admitted (without distinction of sect), but not without hope, however, that, under the best medical advice, some, by God's blessing, may be wholly or partially relieved."

Help. Hope. Home.

The formula, "helpless, hopeless, homeless," however properly it may be applied to incurables generally, is altogether out of place as a description of the fortunate patients who have secured the shelter of the Clapham Road establishment. A less desponding collection of invalid ladies and gentlemen we never saw in our life, and this fact, more than any other, impressed us with the excellence of the system upon which the Home is conducted. Everything about the place is bright and cheerful, though the most scrupulous economy is observed in all the departments. Expense is not spared when necessaries are in question—as, for instance, in the provision of invalid furniture, which is to be seen in every variety; but in the furnishing and appointments of the Home scrupulous neatness and cleanliness, together with the exercise of a simple and effective taste, are apparent, whilst any attempt at luxury has been abandoned. Home comforts are striven after. Each patient who can sit up has his or her own special corner in a particular sitting-room, and meals are served separately, the taste and requirements of each being specially studied. "Weariness," according to Boileau, "was born one day of Monotony," and, so far as is practicable with such sufferers, monotony is not suffered to intrude into the life of the Home. All the sitting-rooms face the Clapham Road, which is a busy thoroughfare, and presents a constantly changing aspect to the onlooker; the rooms are bright with pictures, and adorned for the greater part of the year with flowers, the gifts of many warm-hearted friends in the neighbourhood, whilst there is a constant succession of visitors, amongst whom the clergy and the ladies of the vicinity are not the least welcome. Then there is a spacious garden at the back of the house, with an awning stretched between two great trees in the centre; and here, when the weather is fine and warm, the patients are carried or wheel themselves about in their invalid-chairs. Of the forty-two patients, the majority are able to thus enjoy themselves; and the aspect of the garden, on a hot summer's day, is pleasing enough to touch the heart even of a Scrooge. A love of truth, not a sentiment of gallantry, impels us to say that the happiest and the most cheerful-looking of the patients are to be found on the female side of the house. Women, since the Fall, have always borne suffering better than men; and the resources of the ladies at the Clapham Road Home are indeed wonderful. They seem to be as deft and nimble with their contorted and paralysed fingers as their more favoured sisters in the world outside. The proceeds of their industry they sell every six months or so, and this provides them with a little pocket-money. Needless to say they find many purchasers. Socks and stockings are knitted by the dozen—slowly, it is true; but with perseverance much can be done; whilst antimacassars and such-like household adornments have several busy manufacturers. One lady—the happiness depicted upon whose face it is a pleasure to see—makes excellent presentments of various animals, the calico or linen skin being stuffed with rags. Perhaps the most interesting worker of all is an artist who sits near the window of one of the largest sitting-rooms. On a little tray before her are water-colours, brushes, palettes, and a few cards. The visitor, noticing the bent and cramped hands, whose original shape has been so twisted by rheumatism that they look as if most of the fingers had been cut off at the knuckles, wonders what these artistic implements can possibly be there for. He is soon undeceived. With a pleasant smile the patient produces a birthday or a Christmas card, embellished with carefully coloured and excellently drawn flowers. Astonishment, already great, increases when the information is vouchsafed by the attendant that this lady never had a drawing or painting lesson in her life, and that these prettily designed and executed cards are the result of such studies of nature, and such indefatigable patience, as her sedentary life has rendered possible. Providence, truly, is wonderfully compensatory. Nobody but one afflicted as this poor lady is, would have had the perseverance necessary for success, nor, let us add, the exquisite pleasure which that success seems to give her. Another inmate, a lady of pleasant and refined manners, who welcomes the visitor with a face none the less cordially because she does not rise above a sitting posture, takes an intense delight in exhibiting her "Museum." It is contained in three or four carefully arranged drawers, and every friend who sees it apparently sends a contribution when occasion offers. Curiosities of all sorts are in these little drawers—bits of stone from the Pyramids, specimens of hand-carving from India, paintings from Japan, relics of all sorts from every quarter of the world are displayed and descanted upon, to the delight as much of the narrator as of the interested listener.

Scrooge and the Garden.

The Animal Stuffer, the Artist and the Museum.

*(To be continued.)*

### SCOTCH MIXTURE.

"Mann Peter," said a Scotch quack doctor to his apprentice, "ye maun aye be awfu' cautious in pharmacy. Even I ance made a terrible mistak.' I was attending Mrs. Kittlebody, wha was sair fashed wi' tickdolaroo, an' I was called upon by John M'Fikeit, wha's croon was sae thin o' hair—as weel as sense—that he was ashamed o't, especially as he was coortin' a strappin' young widow that had a fine public-hoose; an' I mixed up baith potions at the same time, an', losh sake, mann, I happened tae gie them ilk ither's medicine! so puir John, rubbing Mrs. Kittlebody's preparation for her tickdolaroo on the tap o' his head, declares he's had a bee in his bonnet ever since; an' Mrs. Kittlebody, rubbin' her jaws wi' the ointment intended for John's bald pow, in less than a fortnicht had a pair o' whiskers the envy o' a' the young men o' the village."

### A NEW DISEASE.

Printers make strange mistakes at times. In looking over the report of a benevolent society, which has been established for many years in Fulham, we observed among the cases which had been relieved, that one was described as "Female, age 34, *abbess* of the neck."

